# Relation between uterine morphology and severity of primary dysmenorrhea

**DOI:** 10.4274/tjod.galenos.2020.26032

**Published:** 2020-07-29

**Authors:** Şenol Şentürk

**Affiliations:** 1 Recep Tayyip Erdoğan University Faculty of Medicine, Department of Obstetrics and Gynecology, Rize, Turkey

**Keywords:** Primary dysmenorrhea, pain, uterine dimensions, visual analog scale score, virgin

## Abstract

**Objective:**

This study aimed to evaluate whether uterine dimensions including uterine volume, uterine shape, uterine length, cervix length, and cervix thickness measurements have a role in the severity of primary dysmenorrhea in virgin girls.

**Materials and Methods:**

Enrollment included 90 virgin girls suffering from primary dysmenorrhea. The girls were divided into three groups according to the severity of dysmenorrhea, which was determined by the visual analog scale (VAS). Patients with VAS scores of 8-10 comprised the severe primary dysmenorrhea group (n=30), 4-7 the moderate primary dysmenorrhea group (n=30), and 1-3 the mild primary dysmenorrhea group (n=30). Uterine characteristics including uterine volume, uterine shape, uterine length, cervix length, and cervix thickness were measured by a high-resolution four-dimensional ultrasound device with real-time capacity. They were recorded to determine if they can be predictors of dysmenorrhea severity.

**Results:**

Girls with severe primary dysmenorrhea were more likely to complain of midline pain as opposed to mild and moderate cases with lateral or diffuse pain. None of the uterine characteristics on ultrasonography examination were significant for predicting the severity of primary dysmenorrhea. There were no significant positive correlations between the dysmenorrhea severity and uterine corpus length, cervix length, and uterine volume degree. Any combination of the measured uterine features was not predictive for determining the severity of dysmenorrhea.

**Conclusion:**

Ultrasonographic measurements of uterine dimensions in virgins have low accuracy for predicting the severity of pain in primary dysmenorrhea.

**PRECIS:** Uterine morphology and severity of primary dysmenorrhea.

## Introduction

Painful menstruation that is not associated with any pelvic pathology is considered primary dysmenorrhea. Although it is a highly prevalent and multifactorial clinical disorder in adolescent girls, the etiology and risk factors of primary dysmenorrhea are unclear^(1)^. Patients with primary dysmenorrhea were more sensitive to other chronic pain than those who have none^(2)^. Subendometrial uterine contraction, sex steroids, local and systemic prostaglandin overproduction, and pain sensitivity may contribute to the clinical background of primary dysmenorrhea. Pain in primary dysmenorrhea is believed to be caused by uterine contractions, ischemia, and prostaglandins^(2,3,4)^. Likewise, it may be influenced by low body mass index, smoking, premature menarche, family history, prolonged menstrual flow, and psychological disorders^(5,6)^. In addition, pelvic pain prostaglandins may increase the contractions of bronchial, intestinal, and vascular smooth muscles, and as a result, they may be related to complaints of nausea, vomiting, and diarrhea that may be seen in primary dysmenorrhea^(7,8)^.

The shapes and dimensions of the uterine corpus and cervix may play a role on the occurrence and severity of dysmenorrhea. One possible mechanism explaining the impact of uterine corpus and cervix length on dysmenorrhea severity is that elongated and thick cervix may pose a mechanical obstruction and/or prolong the menstrual flow, which results in more uterine contractions and eventually dysmenorrhea. The second possibility is based on an opinion that “the bigger the uterus, the more endometrial surface.” This may increase the volume of menstrual blood in the uterine cavity, and the menstrual blood vacuation may come late, which may cause dysmenorrhea. The third possibility is that prolonged exposure of menstrual blood within the uterine cavity and cervical canal may increase the severity of primary dysmenorrhea and could be an etiological factor.

The number of studies evaluating the relationship between dysmenorrhea and uterine shape and diameters are small^(9,10)^. A significant relationship was found between dysmenorrhea severity and uterus corpus and cervix measurements in most of the studies. A recent study by Li et al.^(11) ^showed that the presence of menorrhea and large uterine volume of more than 180 cm^3^ was associated with moderatetosevere dysmenorrhea and urinary tract symptoms.

However, the small number of cases, the small size of the measured parameters, and the differences in the measurement methods necessitated new studies. Therefore, this study aimed to determine whether uterine dimensions such as uterine volume, uterine corpus, and cervical length measurements have a role in the severity of primary dysmenorrhea in virgins.

## Materials and Methods

The study was conducted at the Recep Tayyip Erdogan University Department of Gynecology and Obstetrics with the permission of the Non-Invasive Clinical Research Ethics Board (approval number: 97/2017) between June and October 2017. Study participants included virgin adolescents aged 17 to 25 years who were referred to the gynecology clinic for complaints of dysmenorrhea. Written informed consent was obtained for each patient after they were explained about the study in detail.

### Visual Analog Scale Scores and Participant Grouping

Study participants were selected from cases with regular menstrual periods suffering from primary dysmenorrhea who had no marriage or pregnancy history. Primary dysmenorrhea was defined as suprapubic pain that starts several hours before every menstrual bleeding or on the first day of menstruation and lasts a few hours after the culmination of vaginal bleeding. Pain may spread to the waist, abdominal area, and lateral regions. It peaks with maximum blood flow and continues for 48-72 h^(12)^. Visual analog scale (VAS) scores were used to determine dysmenorrhea severity^(13)^. A study used VAS scores to classify patients with primary dysmenorrhea^(14)^. Participants were then divided into the following three groups based on their level of reported pain: VAS scores of 8-10 comprised the severe primary dysmenorrhea group (n=30), 4-7 the moderate primary dysmenorrhea group (n=30), and 1-3 the mild primary dysmenorrhea group (n=30). For patients in the mild pain group, menstruation is painful but seldom inhibits normal activity, and analgesics are seldom required. Meanwhile, for patients in the moderate pain group, daily activity is affected, and analgesics are required, whereas in the severe pain group, the activity is inhibited, and they are unresponsive to analgesics. In addition to uterine volume, longitudinal and transverse axes of the uterine corpus and cervix were measured. Association between primary dysmenorrhea severity and uterine measurements were calculated.

The age, height, weight, and menarche age of all participants were recorded, and their body mass indices were calculated using the (body weight)/(height)^2^ formula. Each patient’s history of age of menarche, duration of menstrual cycle, period of menstrual cycle, family history of dysmenorrhea, and presence of clinical symptoms accompanying dysmenorrhea (nausea, diarrhea, waist and hip pain, abdominal pain, dizziness, skin rash, fatigue, and irritability) were recorded. Patients with the following pathologies causing secondary dysmenorrhea were excluded from the study: Uterine fibroids, endometriosis, hematometra or hematocolpos, adnexal masses (abscess, ovarian cyst, or hydrosalpinges), vaginal septum or atresia, uterine shape anomalies, pelvic surgery history, pelvic inflammatory disease, urinary or other infections, oral contraceptive or alcohol use and smoking, or nonsteroidal anti-inflammatory drug use in the last 3 days. Girls with premature or late puberty, polycystic ovary syndrome, systemic diseases, and retroverted uterus were also excluded.

### Measurement of Uterine Characteristics

**Figure 1:**
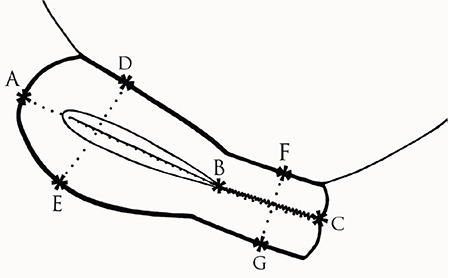
Longitudenal view of uterus during transabdominal ultrasound

**Figure 2:**
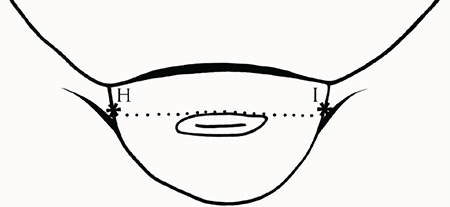
Transvers view of uterus during transabdominal ultrasound

A high-resolution four-dimensional ultrasound device with real-time capacity and a 5-MHz (multi-frequency) transducer (G.E. Voluson 730 Pro 4D Color Doppler Device, USA) were used to measure uterine and cervix lengths. Ultrasonograpy (USG) was carried out in the early follicular phase of the menstrual cycle. The uterine dimensions including corpus and uterine lengths, cervix lengths, cervix thicknesses, uterine volumes, and uterus length/cervix length, corpus length/cervix length, and cervix length/cervix thickness ratios of each participant were calculated and recorded as follows. After bladder filling, the uterine dimensions were measured by transabdominal USG in a supine position. First, the uterine dimensions were measured in gray-scale USG on transverse, longitudinal, and anteroposterior planes. While measuring the longitudinal dimension of the uterus, the fundus and cervix were observed on the same parasagittal plane, and the distance from the top of the fundus to the end of the cervix (AC) was recorded as the uterine length. On the same plane, the distance from the top of the fundus to the beginning of the cervix (AB) was measured and recorded as the uterine corpus length. The distance from the beginning of the cervix to the end of the cervix (BC) was used as the cervix length. The transducer was rotated by 90**°**, and the transvers diameter of the uterus (HI) was measured on the level of the cornual component. On the same plane, the fundus anteroposterior diameter (DE) was measured from the bulgiest part of the fundus, and the cervix anteroposterior (FG) that was measured on the cervical level was used as the cervix thickness (Figures 1, 2). Uterine volume was calculated by assuming that this organ was an ellipsoid with the formula *V*=D1´D2´D3´0.52 [D1= transverse diameter (HI), D2= anteroposterior diameter (DE), and D3= longitudinal diameter (AC)]^(15)^.

### Statistical Analysis

The Number Cruncher Statistical System 2007 (Kaysville, Utah, USA) software was used for the statistical analyses. While analyzing the quantitative data, in addition to descriptive statistical methods (mean, standard deviation, median, frequency, ratio, minimum, and maximum), One-Way analysis of variance was used to compare three or more groups that were normally distributed and the Kruskal-Wallis test for non-normally distributed groups. The qualitative data were compared using the Pearson chi-square and Fisher-Freeman-Halton tests. Significance was analyzed on the levels of p<0.01 and p<0.05.

## Results

Demographic characteristics of each group are shown in Table 1. Both age and menarche age were similar in all groups (p>0.05). Girls with severe primary dysmenorrhea were more likely to complain of midline pain as opposed to mild and moderate cases with lateral or diffuse pain. None of the uterine characteristics on USG examination were significant for predicting primary dysmenorrhea severity. There were no significant positive correlations between primary dysmenorrhea severity and uterine corpus length, cervix length, and uterine volume. Any combination of the measured uterine features were not predictive for determining dysmenorrhea severity (Table 2). Suffering from nausea was found to be significantly higher in girls with severe pain than those in the mild and moderate pain groups (p=0.001 and 0.003, respectively). Significantly low waist and hip pain were noted in girls in the mild pain group compared with those in the moderate and severe pain groups (p=0.001 and 0.010, respectively). Likewise, fatigue and irritability in girls in the mild pain group were significantly lower than those in the severe pain group (p=0.001). The rates of encountering dizziness in girls in the mild pain group were significantly lower than those in the moderate and severe pain groups (p=0.001 and 0.001, respectively; Table 3).

## Discussion

Primary dysmenorrhea is a prevalent problem accompanied by suprapubic pain without the presence of gynecological and pelvic organic diseases. The pathophysiology of primary dysmenorrhea has not yet been clearly explained. When the literature is reviewed, there is not much study investigating the effect of uterine length and volume on dysmenorrhea severity. However, a few studies have tried concluding the relation between uterine dimensions and pain severity by measuring only two markers including uterine or cervix length^(9,10)^, although some only measured the uterocervical angle (UCA) and others evaluated the uterus and cervix length. Unlike other studies, in the current study, we evaluated many parameters, such as uterine and cervical lengths, uterine volume, and uterine and cervix thickness. Longitudinal and transverse axes of the uterine cervix were found to be higher in the severe dysmenorrhea group than in the mild and moderate groups, but the difference between the groups failed to reach a statistically significant difference. In contrast to our findings, Zebitay et al.^(10)^ showed longer cervical length and greater cervical volume in young virgin patients with dysmenorrhea. Dmitrovic et al.^(16)^ found a positive correlation between uterine dimensions, endometrial thickness, and dysmenorrhea severity. The results of this study were not similar to those of Dmitrovic et al.^(16)^. This discordance may be because their control group was composed of patients without dysmenorrhea and the differences in the methods used in uterine and cervix measurements. In contrast to our study, previous studies mostly used transvaginal measurement methods.

A study conducted by Sahin et al.^(9)^ has only tried to conclude dysmenorrheal severity by measuring the UCA. They showed that a narrow anterior UCA is associated with primary dysmenorrhea and disease severity. Contrary to this study, we did not measure UCA, although no significant correlation was found between the stenosis or width of the cervix and the severity of dysmenorrhea. Zebitay et al.^(10)^ reported that the longitudinal and transverse axes of the uterine cervix, as well as its volume, were significantly higher in the dysmenorrhea group than the controls. They also showed a significant positive correlation between the severity of dysmenorrhea and the length of the cervical longitudinal and transverse axes and the volume of the uterine cervix. Our results are incompatible with the results of Zebitay et al.^(10)^, and this discrepancy may result from differences in patient grouping as they only divided their sample into the severe dysmenorrhea and control groups. Their lack of mild and moderate groups may be the main cause of incompatibility. The most important limitation of our study is the absence of the control group. However, this limitation has been minimized because of the subgroup analysis.

In cases of primary dysmenorrhea, various complaints, especially complaints of abdominal, waist, or hip pain, nausea, vomiting, fatigue, irritability, dizziness, and anxiety affect quality of life. In our study, we also aimed to reveal the relationship between pain severity and these complaints in randomly selected cases. In our results, it was determined that the frequency of clinical symptoms such as nausea, fatigue, irritability, and dizziness increased in direct proportion to pain severity.

In this study, we tried to show the correlation between pain severity, clinical symptoms, and uterus dimensions. However, the results of the current study did not support the idea that the uterus dimensions affect dysmenorrheal pain severity. Although we found a positive significant relationship between severity of pain and rates of encountering clinical symptoms, we could not find any single or combined uterine parameters affecting pain severity.

### Study Limitations

Our results may have been affected by the fact that the uterus dimensions of the participants in the study were within physiological limits. Measurement of uterine dimensions by abdominal USG is another important handicap, but virginity was the main reason we used this method. USG measurements combined with magnetic resonance imaging could provide more reliable results.

## Conclusion

Despite several limitations, our study filled an important gap in the literature because this is the first study to evaluate the relationship between uterine dimensions and pain severity in a high number of participants.

## Figures and Tables

**Table 1:**
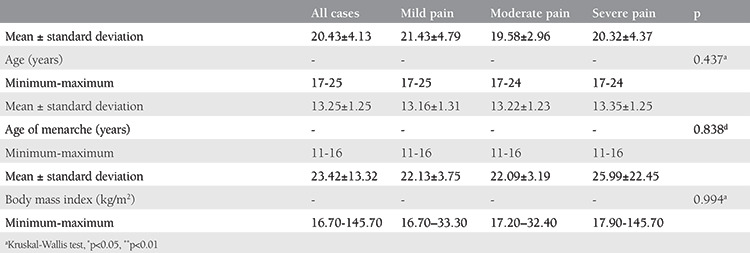
Analysis of demographic characteristics based on pain scores

**Table 2:**
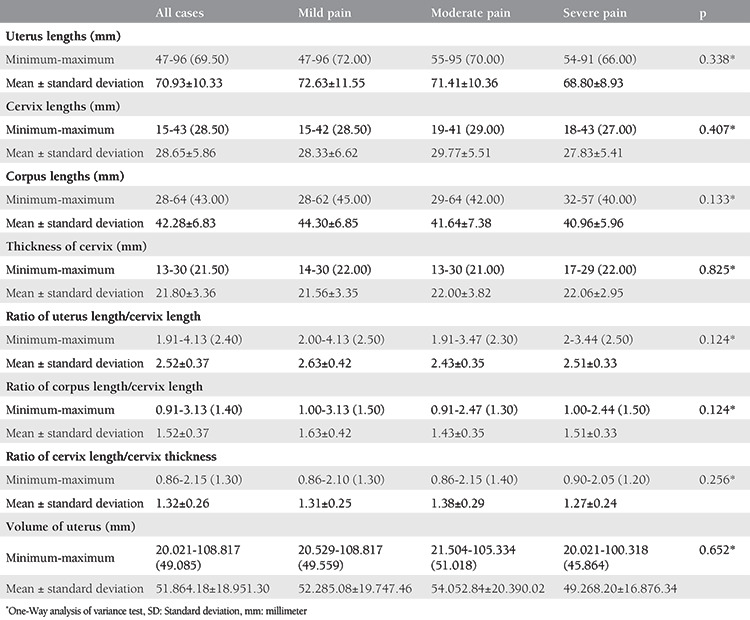
Analysis of anatomic uterus dimensions based on pain score

**Table 3:**
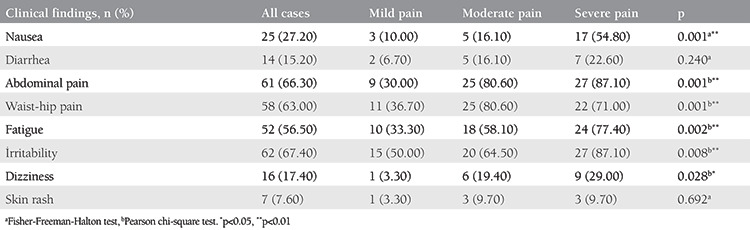
Analysis of descriptive statistics based on pain scores
